# Advances in the Study of Lens Refilling

**DOI:** 10.1155/2020/8956275

**Published:** 2020-08-26

**Authors:** Wen-Wen Zhang, Zheng-Gao Xie

**Affiliations:** Department of Ophthalmology, Nanjing Drum Tower Hospital, The Affiliated Hospital of Nanjing University Medical School, Nanjing 210008, China

## Abstract

The ultimate goal of cataract surgery is to restore the accommodation while restoring distance visual acuity. Different kinds of accommodative intraocular lens (IOLs) and surgical techniques have been suggested to apply during the surgery, but they showed poor postoperative accommodation. It is possible to achieve this goal by refilling the lens with an injectable polymer. We present a summary of the existing materials, methods, results, and some obstacles in clinical application that remain of lens refilling for restoration of accommodation. Two main problems have restricted the clinical application of this technique. One was the formation of postoperative secondary capsule opacification and the other was the different accommodative power after surgery.

## 1. Introduction

The modern cataract surgery has gradually changed from traditional restorative surgery to refractive surgery, which puts forward high requirements on the surgical techniques and intraocular lens (IOL). All kinds of surgical techniques and intraocular lens available have not achieved the ultimate goal of preserving physiological accommodation. The ideal intraocular lens should be similar in size, shape, position, and optical properties to crystalline lens and possess four physiological properties of human natural lens: transparency, optical performance, barrier function of anterior and posterior segments, and regulatory capability [1]. The injected IOL attempted to form a lens with physiological accommodation in situ. During the operation, the nucleus and cortex of the lens were removed through minute anterior capsule opening, and then the material was injected into the capsule. The material solidified into the shape of lens according to the shape of the capsule. Thus, the injected lens could obtain a lens with the same regulatory potential as a normal lens while maintaining the integrity of the ciliary muscle, suspensions, and capsular membrane [2]. It may be a promising choice to refill the capsule with elastic polymer.

## 2. Injection Materials

In order to achieve the desired result of the injected crystalline material, the material should meet the following requirements: (1) easy injection into the capsule and no obvious leakage under different intraocular and extraocular pressure; (2) no immunogenicity, good histocompatibility, and no obvious eye inflammation after injection; (3) good optical properties, light transmittance to reach more than 96%, refractive index of about 1.420, and absorption of ultraviolet light effectively; (4) no weightlessness and swelling occurring; (5) not being biodegradable and ability to rapidly polymerize in liquid form to an elastic gel state [1, 3]. The materials that had been researched and could be used as injectable intraocular lens are introduced as follows.

### 2.1. Silicon and Silicon Containing Polymers

As early as the 1860s, Kessler's research group first reported that liquid silicon was used as an injectable lens material [4, 5]. Under normal temperature, the material would change into an elastic semisolid viscous substance and form a physical gel. However, the gel state of the material had less regulation function, thus abandoning it. Agarwal's et al. [6, 7] and Parel's et al. [8, 9] research groups proposed to use silicone as the injection lens material. Silicon was an organic polysiloxane with strong hydrophobicity, poor affinity with the body after implantation, and long curing time (about 12 hours) leading to leakage easily, thus causing a series of serious complications. In addition, low refractive index (1.400) and poor elasticity also lead to its abandonment.

In the 1990s, Nishi et al. proposed to implant silicone gel balloon [10, 11] or plug [12] to seal the capsule, so as to create a closed bag, which could solve the material leakage problem. Because of the low refractive index (1.401) and weak regulation changes of silicone, some material of more suitable refractive index should be injected into the capsule. Nishi's team has made a series of studies on this and proposed using a mixture of two silicone compounds, polydimethylsiloxane as the main component and hydrogen polysiloxane as a cross-linking (1 : 1 v/v) [13–16]. The mixture had a suitable polymerization time (2 hours), good optical performance close to the normal lens, and excellent light transmittivity and biocompatibility. However, there were still some problems; for example, the refractive index could not meet the requirements (1.405) and the incidence of postoperative capsular opacification was high [13]. In the team's latest studies, polymers were injected between two IOLs to solve both refractive index deficiencies and postoperative capsule opacification [17, 18]. A foldable silicone accommodative membrane IOL was made of silica gel, and the refractive index of silica gel was 1.410. Therefore, in ray tracing, the refractive index of the membrane IOL in air was +19.5D. And the refractive index of injected silicone polymer was 1.397 [18].

Another kind of silicone polymer was proposed in the studies of Koopmans et al. [19–21]. The polymer needed 70 minutes to polymerize at 20°C and 30 minutes to polymerize at 35°C and had a 0.8 kPa Young's modulus finally. For comparison, a 20-year-old human lens' Young's modulus was 1.0 kPa [22]. Young's modulus of the material maintained the same for 100 days. And the refractive index was 1.428.

In general, silicon-containing polymers had good optical properties, good biocompatibility, and stable structure, which can exist in the eye for a long time. In view of the above advantages, silicon-containing polymers had certain clinical application [23–25], but their shortcomings could not be ignored, such as strong adhesion prone to crystal surface opacity [26–29].

### 2.2. Photopolymerization Compounds

In order to overcome the long polymerization time of silicon containing polymers, some researchers proposed photopolymerization compounds. Hettlich's research team developed a liquid monomer, which can be polymerized in situ by exposure to blue light with a wavelength of 400–500 nm in only 20 seconds [30–32]. Moreover, the substance itself had the characteristics of contact inhibition, which significantly reduced the incidence of the rate of secondary opacification in rabbits, especially when the capsule refilled completely [31]. However, the refractive index was 1.532, and there was no elasticity after polymerization, so it was abandoned. Han et al. suggested that 25% poloxamer hydrogel seemed to be a choice of artificial crystal for injecting intraocular lens materials [33]. The hydrogel induced irreversible gelation by photoinitiator and ultraviolet irradiation [34]. It had good light transmittance and no obvious inflammation and capsular opacity (half a year), but it had a low refractive index (1.36) and the possibility of melting and leaking [33]. Groot et al. proposed a hydrogel, which was cured under blue light initiator and blue light irradiation [35]. The light transmittance of the hydrogel was similar to that of 25-year-old young people. The refractive index after curing was 1.42, but the solution viscosity before curing was too low to be easily injected into the capsule [35].

As lens refilling materials, photopolymeric compounds had their certain shortcomings, but the proposal of this material provided a new idea for the selection of other materials. Hao and others put forward a kind of polysiloxane that could be polymerized by photoinduction [36, 37]. The functionalized polysiloxane was a macromonomer, which could achieve the targeted mechanical properties of soft gel through controlling the molecular weight and cross-linking density of itself. Likewise, the viscosity before curing was also tailored by manipulating the molecular weight of the macromonomer. The refractive index also could be precisely controlled by adjusting the aromatic ratio in the macromonomer. The material was solidified in situ under blue light irradiation for 5 minutes under the condition of photoinducer, and a soft gel with light transmittance over 95% in the visible wavelength range was obtained. The material had good biocompatibility and could absorb ultraviolet light below 400 nm.

### 2.3. Other Compounds

Hettlich and Asiyo-Vogel proposed to insert a balloon made of polydimethylsiloxane into the porcine lens capsular bag, then injected 2% methylcellulose or silicone oil into the balloon to observe the effect of accommodation, and finally abandoned the experiment because of the cumbersome surgical methods [38]. Aliyar et al. put forward the reversible disulfide copolymerization hydrogel [39]. The hydrogel was nonexothermic, monomer toxic, and curing in situ within 5 minutes at PH = 7 without leakage. Its Young's modulus could be changed by altering the concentration and thiol content. Lee et al. used a 4-armed PPO/PEO-hydrogel curing rapidly in situ under the action of horseradish peroxidase [40]. The gelation time could be controlled between 20 seconds and 2 minutes, and Young's modulus could be controlled between 1 and 43 kPa.

## 3. Surgical Technique

The procedures of lens refilling were similar to traditional phacoemulsification combined with IOL implantation. The general experiment steps were as follows: general anesthesia and mydriasis, doing a transparent corneal incision of 3.0 mm, injecting viscoelastics into the anterior chamber, making a side incision (early by a cannula and then a paracentesis knife), anterior lens capsule capsulorhexis, phacoemulsification in the capsule, cortex absorption, injection of the IOL material, closure of the anterior capsule, viscoelastic absorption, and sealing the corneal incision.

Compared with the traditional cataract surgery, the difference of this surgery was mainly in the preparation and sealing of the anterior capsule and material injection. Continuous curvilinear capsulorhexis (CCC) was commonly used in traditional cataract surgery, but the capsule opening was too large, which was easy to cause leakage of the refilling material. In order to avoid the problem, capsular opening should be as small as possible and should have smooth opening edge and strong tensile resistance. Many attempts had been made by researchers to make the lens refilling experiment successful. Now we will introduce the surgical techniques in the documents as follows.

**Figure 1:**
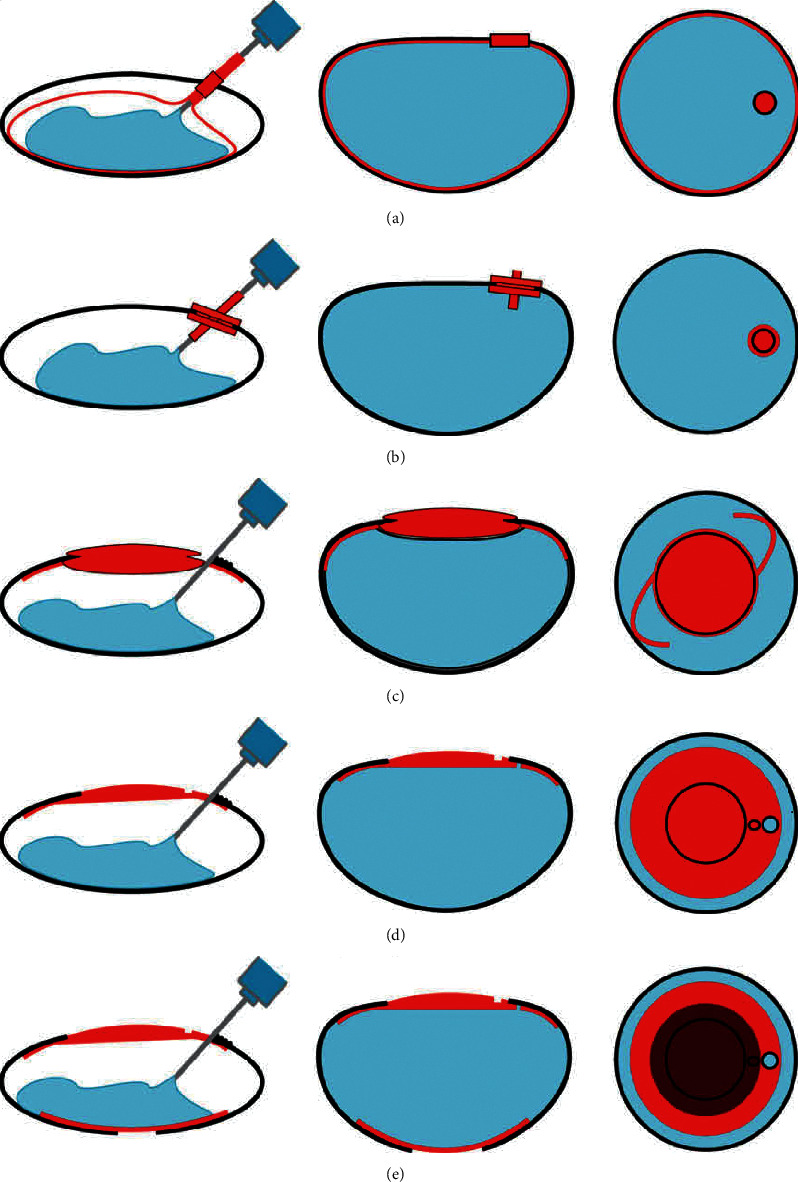
Schematic representation of lens refilling technology in animal experiments. The chart depicts the filling technique (left) and sagittal (middle) and frontal (right) views of filled capsular bag with injectable polymers (blue) and implanted devices (red). (a) Endocapsular balloon by Nishi [14, 41]. (b) Capsule sealing plug by Nishi [12, 13]. (c) Optic plug by Nishi [17]. (d) Accommodative IOL by Nishi [17]. (e) Accommodative IOL with posterior CCC optic by Nishi [17].

**Figure 2:**
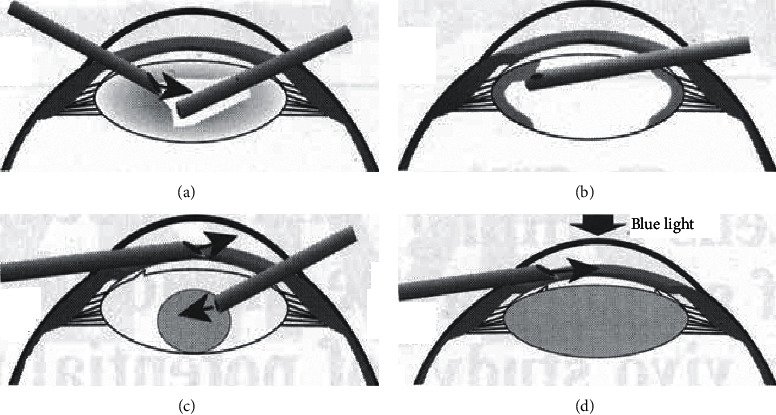
(Hettlich) surgical technique: bimanual phacofragmentation and cleaning of the capsular bag (a, b); refilling of the capsular bag (c); endocapsular polymerization (d).

In Nishi's researches, following methods were used: (1) making a small “buttonhole” or “dumbbell” opening in the anterior capsule or a minicircular opening with a diameter of 1.5 mm∼1.8 mm and simultaneous preservation of capsular integrity, implanting an inflatable balloon after endocapsular phacoemulsification, extracting gas from the balloon, and injecting a liquid silicone polymer through a delivery tube [11, 12, 14, 16] (Figure 1(a)); (2) placing a silicone plug in the CCC area to prevent leakage of the injecting material on the basis of microcapsule and balloon [13] (Figure 1(b)); (3) making an anterior capsule opening with a diameter of 3.5 mm∼4.0 mm, a routine phacoemulsification, a sharp edge IOL implanted just before the posterior capsule, a disc-shaped anterior accommodative IOL (AC-IOL) to prevent leakage and provide a certain optical capability, and injecting silicone polymer between the two lenses [17, 18] (Figures 1(c)–1(e)).The AC-IOL had been improved: the diameter was 9.0 mm, with thick edge and thin center; the anterior and posterior radii of curvature were 15.5 mm and 9.0 mm, respectively, and the edge had an injection hole of 0.8 mm, through which a mixture of silicone polymer was injected into it with a 22G needle [18].

There is some little difference in other's researches of the surgical technique. Koopsmans et al. used a 27G needle to puncture the anterior capsule and made a 1.0 mm∼1.5 mm size circular capsule opening with the Utrata pliers. Then they manually sucked out the pig cadaver lens using 18th caliber or 20th caliber cannula, sealed the capsule opening with a 2.7 mm diameter silica gel plug, and then injected the material [19, 42]. Hettlich et al. made a smaller corneal incision and used a bimanual phacofragmentation through two opposing 1.0 mm corneal incisions and two 1.0 mm diameter peripheral anterior capsule openings. They removed the capsular contents completely by using curved needles and suction tips [30–32, 43] (Figure 2). During the operation of Han et al., a 2.5 mm clear corneal incision at 12 : 00 was made for phacoemulsification [33, 34]. A larger lateral incision than normal was made at 3 : 00 and the diameter of the capsulorhexis was about 1.5–2.0 mm. The procedure of Tahi et al. was based on Tahi's research [44]. In addition to the aforementioned surgical techniques, Hara had developed a microtrephine that could be used to create 0.9 mm or 0.5 mm anterior capsular openings [44, 45]. The techniques proposed could be used to make the anterior capsule and remove cataract successively.

## 4. Postoperative Effect

Lens, suspensory ligament fiber, and ciliary muscle constitute the essential components for eye accommodation. During the regulation, the thickness and shape of the crystal could be precisely adjusted to see the target object [46, 47]. The lens was regulated mainly by changes in the anterior curvature [19, 46, 47].

Nishi et al. used balloon inserted into capsule in rabbit eyes and pig cadaver eyes and filled silica gel in the balloon to obtain −1.0D accommodation power [11]. Considering the rabbit lens accommodation range was only 2.0D, the same experiment was also done on primates, and one of three primates obtained 6.0D accommodation [14]. Considering that postoperative diopter was mainly determined by the refractive index of injected materials and the anterior capsule curvature, which depended on different filling degrees, the more the capsular bag was filled, the steeper the curvature of the anterior was and so the greater the refraction was. Then their team repeated the same experiment in pig eyes: the average curvature of anterior capsule was 6.50 ± 0.07 mm at 17 h and 6.54 ± 0.04 mm at 42 h after surgery [12]. The anterior capsular curvature changed with the applied tension to the suspensory ligament at 17 h after surgery and was no longer affected by the tension applied to the suspensory ligament at 42 h after surgery. Their study also found that there would be different accommodation with the different injection amount in the capsular bag. The filling content was 45%, 55%, 75%, and 95%, respectively, and the corresponding regulatory power obtained was 3.2 ± 0.5D, 6.1 ± 1.8D, 4.8 ± 0.8D, and 2.8 ± 1.3D, respectively. Therefore, it was believed that moderate (60∼70%) refilling would produce a greater regulatory amplitude. Repeating the experiment on rabbit eyes, the filling volume was about 66% and the postoperative refractive power was 0.2D ± 1.5D [16]. The postoperative adjustment amplitude was 1.0∼4.5D, averaging 2.3 ± 1.3D, when the experiment was repeated on eight rhesus monkeys, and the preoperative accommodation amplitude (5.75∼11.25D) was only partially recovered [13]. Recently, Nishi's team injected refilling material between the AC-IOL and the sharp-edged IOL (Figure 1(d)) [18]. The filling content was equivalent to 65%, 80%, and 100% of the pouch, respectively. The accommodation power obtained was 2.56 ± 0.74D, 2.42 ± 1.0D, and 2.71 ± 0.63D, respectively. Therefore, this study suggested that regulatory amplitude did not depend on the filling degree of the capsular bag, which was different from directly injecting materials into capsular bag. The result might be due to the implantation of the AC-IOL. The specific mechanism needs to be further studied.

Sakka and associates refilled the endocapsular silicone balloon by an organosilicon mixture [48]. In the refilled eyes, the average anterior chamber depth (ACD) was 0.5 mm and the average maximal myopic change was 6.74D after topical application of 4% pilocarpine. The research of Koopmans and associates on pig cadaver lens showed that the refractive power obtained by filling the capsule with silicone polymer was related to the thickness and volume of the filled lens (0.54 ml/*D* and 0.04 ml/*D*, respectively), and the spherical aberration of pig lens changed from natural negative spherical aberration to positive spherical aberration after filling [20]. Then, the team conducted research on the spherical aberration change and found that the spherical aberration change was mainly caused by the change of refractive index from the gradient refractive index of natural state to the uniform refractive index, rather than the change of lens profile [21]. Koopmans's team filled the capsule with a silicone polymer in nine adolescent rhesus monkeys [42]. The maximum postoperative accommodation amplitude was 6.3D, and the accommodation amplitude remained stable at ±4D during the follow-up period in two surgically treated eyes. In the research of Hao and associates, lens stretching was performed on postmortem tissues from the eyes of nonhuman primates [36]. The experiment showed that the refilled lens maintained on average 10.36D ± 3.56D (uncured polymer) and 8.37D ± 2.33D (cured polymer) accommodation, respectively, compared to 14.04D ± 3.88D accommodation for natural young primate lens. In other words, the refilled lens maintained on average of 73.9% and 61.9% of accommodation amplitude of the natural young primate lens using uncured and cured polymer, respectively [36]. The change of refraction after injecting a 4-armed PPO/PEO-hydrogel into the capsule was +0.83D, compared to 0.42D preoperatively, which might indicate the maintenance of accommodation amplitude [40].

## 5. Existing Problems in Lens Refilling

There were still some problems in lens refilling (Table 1). Generally speaking, the main problems were leakage of refilling materials, insufficient accommodation, and secondary capsule opacification (both anterior and posterior capsule).

### 5.1. Leakage of Refilling Materials

The leakage of injection materials could lead to corneal edema, neovascularization, iris atrophy, and posterior adhesion. The risk of leakage caused by direct injection of refilling material into capsular bag was significantly higher than that of injection into the endocapsular balloon. To prevent leakage of injection materials, some solutions had been developed (Table 1). Nishi's team has come up with three solutions: (1) endocapsular balloon [10, 11, 41], (2) the method of silicone gel plug sealing the anterior opening [12], and (3) the method of foldable membrane IOL (i.e., AC-IOL), sealing the anterior opening [17, 18]. The endocapsular balloon method and silicone plug method have also been studied in other research teams, which significantly reduced leakage [19, 42]. However, the two methods still had their own problems. For example, the balloon method might have different shapes of the balloon and capsular bag, thus affecting the postoperative accommodation amplitude. The membrane IOL recently proposed by Nishi proved to be effective but needs further study. There were some other studies that use photopolymerization compounds as refilling materials, which not only reduced leakage through the short solidifying time, but also caused little capsule opacification, but the degree of opacification was not quantitative [30–35].

Although removing leaking material was relatively simple, both intraoperatively and postoperatively, and a small amount of leakage of injected material would not cause serious complications, in general, the existing methods to prevent material leakage have not been proved to be simple and effective in human.

### 5.2. Accommodation Power

The refilling materials and the operation methods mentioned above were different, but the accommodation range obtained was limited. The reason was related to insufficient refractive index of the refilling materials (1.36∼1.428) and the secondary capsule opacification to be described later. According to the ideal refractive index of the refilling material (1.420 ± 0.002), there were two materials mentioned above meeting the refractive index requirements. One was a hydrogel with a refractive index of 1.42 [32] and the other was a mixture composed of silicone polymers with a refractive index of 1.428 [19]. The transmittance of the hydrogel proposed by Groot et al. after copolymerization was comparable to that of natural lens before 25 years of age. However, the viscosity of the hydrogel before copolymerization was too low, so it was difficult to manufacture the hydrogel lens in the capsular bag [32]. Koopmans et al. applied the mixture composed of silicone polymers with a refractive index of 1.428 to nine adolescent rhesus monkeys, which were divided into two groups [19]. In the first four monkeys (group A), only one monkey could measure refraction and the three other monkeys could not get refraction measurement because of postoperative inflammation and capsular opacification. In a second group of five monkeys (group B), all the monkeys were given a treatment to delay the development of capsular opacification and the maximum accommodative amplitude was 6.3D.

The refractive index of the above two experimental materials met the requirements, but the accommodative power obtained only accounted for a part of preoperation, which may be related to the loss of intracapsular regulatory function. Intracapsular regulation refers to the involvement of lens fiber cells in regulation; that is, the fiber itself does not move, but the content of the fiber moves during the regulation process. The specific mechanism is not clear, and the indirect evidence of its existence can be found in some literatures [50, 51]. If there is such accommodation in the capsule, it is impossible to completely restore the whole accommodation power only by refilling the lens with artificial materials. However, considering that the accommodation range of 3D is sufficient for the postoperative near vision, it is not necessary to recover the full accommodation range of lens for humans. But it is known that approximately twice the accommodation amplitude is required to support reading over a longer period of time without visual fatigue [52].

### 5.3. Secondary Capsular Opacification

Elasticity and transparency of the capsule were essential factors to the success of lens refilling. After cataract surgery, there were two ways in which lens epithelial cells (LECs) proliferated and caused secondary capsular opacification: fibrosis and regeneration. The former was rarely seen in clinical practice and regeneration secondary capsular opacification can be treated with YAG posterior capsulotomy. However, in the case of lens refilling, posterior capsulotomy carried a risk of material leakage, so it might be difficult or even impossible to treat regenerative cataract after lens refilling. This made secondary capsular opacification a major obstacle to experimental lens refilling (Table 1). Therefore, prevention of secondary capsular opacification was of great significance for lens refilling. We replaced the posterior capsular opacity (PCO) with secondary capsular opacification because the former generally referred only to posterior capsular opacity.

Nishi's group pointed out that adequate filling of capsule might inhibit the proliferation and migration of LECs as early as in a study conducted in 1992 [14], which was consistent with the reported results of photopolymerization compounds (a significant decrease in PCO occurred after surgery due to the contact inhibition of the hydrogel itself [30–32]). Nishi's team then used a sharp-edged IOL implanted just in front of the posterior capsule and made CCC in the center of the posterior capsule in some experimental animals intraoperatively in order to reduce the secondary capsular opacification [17]. Five rabbits with CCC in the center of the posterior capsule had no PCO 5 to 8 weeks after operation. In the five rabbits without intraoperative treatment, two had no PCO and three had only mild to moderate PCO. The results showed that the use of sharp-edged IOL alone just before the posterior was not enough to eliminate capsular opacification, and posterior CCC (PCCC) was a good method to ensure the transparency of the central part of the optic axis. However, how to carry out PCCC without leakage of materials might be a difficult problem for us to study.

Koopmans et al. applying cycloheximide and actinomycin D treatment of the capsular bag during surgery effectively delayed the formation of secondary capsular opacification [42]. 40% of the treated monkeys maintained the accommodation range at about 4D during the follow-up period and 60% of the monkeys at 37 weeks after the surgery, although refractive measurements could be performed and the amplitude of accommodation almost decreased to 0D. Further study by Koopmans's team found that the use of cycloheximide alone did not alleviate secondary capsular opacification, whereas actinomycin D treatment of rabbit capsular bag for 5 minutes significantly reduced capsular opacification in three months after surgery [49]. However, because some side effects (corneal opacity, etc.) had been produced in some experimental rabbits and not all rabbit capsules treated with actinomycin D did not develop secondary capsular opacification, new drugs and improved experimental methods need to be developed.

There have been many reports on the prevention of PCO by surgical techniques and various drugs in modern cataract surgery: (1) the incidence of PCO using intraocular lens with sharp edge was significantly reduced [53]; (2) the incidence of PCO using intraocular lens with different materials (polymethyl methacrylate, hydrogel, hydrophobic acrylate, and silicone) had little difference, but the PCO index of hydrogel was higher than that of other materials, while the PCO index of silicone was lower than that of other materials [53]; (3) adequate polishing of the capsule during the surgery had a certain effect on reducing PCO, but it was not enough to completely remove LECs to prevent PCO [54–58]; (4) there were various anti-inflammatory treatments during and after the surgery, except for immunotoxin (MDX-A); the others did not reduce the incidence of PCO [53].

## 6. Conclusions

Although lens refilling has made some progress in animal experiments, there are still some problems that cannot be solved perfectly. Overall, secondary capsular opacification remains to be the major obstacle, as it results in decreased elasticity and clarity of the capsule. It is believed that, with the further development of lens refilling materials and the progress of the capsular opacity treatment after cataract surgery, lens refilling is expected to be an ideal method for cataract treatment.

## Figures and Tables

**Table 1:** Lens refilling experiments published in animals.

Year	Author (citation)	Animal model	Implant material	Procedure	Main findings	Problems
1989 [11] 1992 [14]	Nishi	Rabbit, pig, and monkey	Silicone, balloon, balloon filled with silicone polymer	Endocapsular balloon	No leakage, little accommodation in rabbit eyes (1D), 6.0D of accommodation in a single primate eye	Undersized balloons result in residual hyperopia and progressive decrease in accommodation amplitude with fibrosis
1992 [30] 1994 [31] 1995 [32]	Hettlich	Rabbit and pig	Acrylate copolymer	Two 1.2 mm capsulotomies, endocapsular polymerization with ultraviolet light	No leakage, no obvious inflammation, secondary opacification appeared less especially in completely refilling	Iris irritation, capsule refilled incompletely leading obvious secondary capsule opacification
1996 [48]	Sakka	Monkey	Balloon filled with an organosilicone mixture	Endocapsular silicone balloon	6.74D of average maximal myopia change	Secondary capsular opacification
1996 [38]	Hettlich	Pig	Balloon filled with 2% methylcellulose or silicone oil	Ellipsoid balloons made of polydimethylsiloxane; enlarge the tunnel incision to 4.5 mm; leave capsule complete	Not mentioned	Complicated surgical procedures
1997 [12] 1998 [13] 1998 [16]	Nishi	Pig monkey rabbit	Silicone mixture that polymerized in vivo in 2 hours	Plug for sealing 1.5 mm rhexis, attempt different levels filling	No leakage, moderate filling accept most accommodation	Rapidly developing secondary capsular opacification
2003 [33] 2005 [34]	Han	Rabbit	25% poloxamer hydrogel	Plug for sealing 1.5–2.0 mm CCC, 2.5 mm clear corneal incision at 12 : 00, a larger side port at 3 : 00	No apparent ocular inflammation or posterior capsule opacification	Low refractive index, no postoperative accommodation results in primates
2005 [39]	Aliyar	Pig	Acrylamide hydrogels containing disulfide bonds by free radical polymerization in aqueous ethanol	Not mentioned	The moduli ranged from 0.27 to 1.1 kPa No leakage, no heat release, and no toxicity	Lack of in vivo experiment, lack of accommodation measurement
2003 [19] 2006 [42] 2011 [49]	Koopmans	Monkey	Silicone polymer	2.7 mm plug for sealing 1–2.0 mm CCC, surgically treated: cycloheximide and/or actinomycin D injected in capsular bag for 5 min	6.3D accommodative amplitude after surgical treatment; capsular opacification reduced the accommodation, a safe application of actinomycin D	Secondary capsular opacification cannot be completely prevented by actinomycin D
2004 [20] 2007 [21]	Koopmans	Pig	A two-component silicone polymer	Plug for sealing 1–2.0 mm CCC, measure the thickness, the focal length, and the spherical aberration after the initial lens refilling	Increased lens filling volume associated with decreased accommodative amplitude (0.04 ml/D, 0.54 mm/D), the positive SA changed to negative after refilling	Lack of in vivo experiment
2008 [17] 2014 [18]	Nishi	Rabbit. pig, and monkey	Silicone polymer	Foldable silicone optic for sealing 5.0 mm rhexis, sharp-edged IOL implanted before posterior capsule, +/- optic for posterior rhexis	Prevention of central ACO and PCO with posterior optic, around 2.5D of accommodation obtained independent of filling degrees of the capsular bag	Inflammation
2010 [36] 2012 [37]	Hao	Rabbit fresh cadaver	Functionalized siloxane macromonomer	1.3 mm peripheral capsule hole with diathermy, 20G cannula	Over 60% accommodation in rabbit eyes, more than 100% accommodation in fresh cadaver eyes	Secondary capsular opacification started at 7 days after surgery and strong lens regeneration occurred at about 6 weeks
2014 [40]	Lee	Rabbit	4-armed PPO/PEO-phenol	Synthesized by horseradish peroxidase	Refraction after lens refilling indicating the maintenance of accommodation amplitude	Lack of further studies in primates

CCC = continuous curvilinear capsulorhexis; SA = spherical aberration; ACO = anterior capsule opacification; PCO = posterior capsule opacification.
